# Analysis of the Relationship between Estradiol and Follicle-Stimulating Hormone Concentrations and Polymorphisms of Apolipoprotein E and LeptinGenes in Women Post-Menopause

**DOI:** 10.3390/ijerph13060543

**Published:** 2016-05-28

**Authors:** Aleksandra Rył, Andrzej Jasiewicz, Anna Grzywacz, Grażyna Adler, Karolina Skonieczna-Żydecka, Iwona Rotter, Olimpia Sipak-Szmigiel, Bogdan Rumianowski, Beata Karakiewicz, Anna Jurczak, Miłosz Parczewski, Anna Urbańska, Marta Grabowska, Maria Laszczyńska

**Affiliations:** 1 Department of Histology and Developmental Biology, Pomeranian Medical University, Szczecin 71-210, Poland; ryl.ola@gmail.com (A.R.); bogdan.rumianowski@gmail.com (B.R.); grabowska.marta.anna@gmail.com (M.G.); maria@laszczynska.pl (M.L.); 2 Department of Psychiatry, Pomeranian Medical University, Szczecin 71-460, Poland; andrzej.jasiewicz@gmail.com; 3 Department of Gerontobiology, Pomeranian Medical University, Szczecin 71-210, Poland; gra2@op.pl (G.A.); karzyd@sci.pum.edu.pl (K.S.-Z.); 4 Department of Rehabilitation Medicine, Pomeranian Medical University, Szczecin 71-210, Poland; iwona.rotter@pum.edu.pl; 5 Department of Obstetric and Gynecological Nursing, Pomeranian Medical University, Szczecin 71-210, Poland; olimpiasipak-szmigiel@wp.pl; 6 Department of Public Health, Pomeranian Medical University, Szczecin 71-210, Poland; karabea@pum.edu.pl; 7 Department of Nursing, Pomeranian Medical University, Szczecin 71-210, Poland; jurczaka@op.pl; 8 Department of Infectious, Tropical Diseases and Immune Deficiency, Pomeranian Medical University, Szczecin 71-455, Poland; milosz.parczewski@pum.edu.pl (M.P.); urbanska@pum.edu.pl (A.U.)

**Keywords:** menopause, hormones, apolipoprotein E, leptin

## Abstract

*Background*: Menopause is the permanent cessation of menstruation due to loss of ovarian follicular activity. A review of the available literature indicates that correlations between the changes that take place in a woman’s body after menopause and different genetic variants are still being sought. *Methods*: The study was conducted in 252 women who had completed physiological menopause. The women were divided into groups according to the time elapsed since menopause. The total concentrations of estradiol and follicle-stimulating hormone were determined by means of electrochemiluminescence. The apolipoprotein E *(APOE)* and lepitn *(LEP)* genotypes were determined by real-time PCR and polymerase chain reaction–restriction fragment length polymorphism, respectively. *Results*: We observed that people with the *APOE3/E3* genotype entered menopause insignificantly later compared to other genotypes. Additionally, in the group of patients with the *APOE3/E3* genotypes, differences in the E2 concentration were significantly related to the time since their last menstruation. There is no association found in the literature between these polymorphisms of the *LEP* gene and hormones. *Conclusions*: To date, attempts to formulate a model describing the association between E2 and FSH concentration with the polymorphisms of various genes of menopause in women have not been successful. This relationship is difficult to study because of the number of nongenetic factors. Environmental factors can explain variation in postmenopausal changes in hormone levels.

## 1. Introduction

According to the 1996 definition of the World Health Organization (WHO), menopause is the permanent cessation of menstruation due to loss of ovarian follicular activity [1,2]. Because of the substantial ontogenic diversity of women at the age when their menstruation disorders, climacteric symptoms, and the final menstruation occur, a system of uniform classification of hormonal, biological, and clinical changes called STRAW (Staging Reproductive Aging Workshops) was suggested in 2001 [3]. The time of final menstruation depends mainly on genetic [3,4,5,6] factors, but also on iatrogenic factors in cases where women have undergone chemotherapy, surgery, or radiation therapy of the ovarian region [6].

Estrogen deficiency results in the increased secretion of insulin by the pancreas, metabolic disorders, the development of obesity, and insulin resistance [7,8,9,10,11]. Our previous research studies have shown the presence of receptors for FSH and LH [12] as well as α estrogen receptors [13,14].

Leptin is a protein consisting of 167 amino acids that is coded by the *LEP* gene mapped on chromosome 7q31.3 [15,16]. The gene consists of three exons separated by two introns. The coding region is located in exons 2 and 3 [17]. Within the promoter region of this gene, a few sequences controlling its expression have been found [17]. Expression control occurs mainly in the cells of white adipose tissue, and its extent is dependent on the location of this tissue in the organism [15,16]. Numerous polymorphisms of the *LEP* gene and its receptor (coded by the *LEPR* gene) have been identified. One of the most commonly described polymorphisms of the *LEP* gene is the substitution of cytosine (C) for adenine (A) at position -2548 within the promoter region, located above the ATG transcription start site [18]. It has been demonstrated that polymorphism -2548 G<A is related to the risk of menopause, osteoporosis, and obesity, as well as cancer of the breast and endometrium [18,19].

Changes in particular hormonal actions occur after menopause, and have an impact on women’s health and psychosocial life [20]. A gene of great importance to the development of neurovegetative changes in the brain is apolipoprotein E (*APOE*), located on chromosome 19 (region 19q13.2) [21,22].

Apolipoprotein E (*APOE*) is a polymorphic protein with three isomorphic forms, including *APOE2*, *APOE3*, and *APOE4*, coded by three allelic arrangements of the *APOE* gene—namely, *E2*, *E3*, and *E4* [23,24]. The most common allele in Caucasian women is the *APOE3* allele, which is found in 78% of cases. The *APOE4* allele is found in 14% of the population, while the *APOE2* allele occurs in 8% of the women covered by the previous study [25].

*APOE* is synthesized in a range of tissues, including the brain, liver, spleen, adrenal glands, and kidneys. The primary site of the *APOE* mRNA expression is the liver, secreting approximately 75% of this protein that circulates in blood plasma, with the brain being the second key secreting tissue [25]. The *APOE4* isoform protects neurons from oxidative stress less actively, with a less efficient decrease in microglia and astroglia activation, and has been associated with severe inflammation [26,27]. The presence of the *APOE 4* allele has also been related to amyloid beta accumulation in the brain, which is of great importance in the pathogenesis of Alzheimer’s disease [28,29]. Furthermore, the *APOE3/E4* and *APOE E4/E4* genotypes have been associated with better memorization. On the other hand, the *APOE E3/E3* genotype is related to higher concentrations of prolactin and increased psychomotor speed [30]. It was found that polymorphic variants of the *APOE* gene can have an impact on hormones, including estrogen in the central nervous system [31].

A review of the current literature shows that correlations between the changes that take place in a woman’s body after menopause and different genetic variants are still being sought. In particular, the literature contains few articles that directly address the relationship between the polymorphisms investigated here and menopause. The aim of this study was to examine the association between estradiol and follicle-stimulating hormone concentration with polymorphisms of *APOE* and *LEP* genes in postmenopausal women.

## 2. Materials and Methods

The study was conducted in 252 women who had completed physiological menopause and who did not use menopausal hormone therapy. The mean age of the cohort was 59.1 (±6.4). The women were divided into three groups, according to the time that had elapsed since menopause. Groups A, B, and C included women who were respectively <5, 6–10, and over 10 years after menopause.

Patients with active and uncompensated hyperthyroidism, as well as an active alcohol dependency or active cancerous disease, were excluded from the study. Those patients who were being treated with antipsychotics, antidepressants, or steroids were also excluded from the study. Informed consent was obtained from all participating patients.

Venous blood was collected from the ulnar vein to determine hormonal and genetic parameters. Serum was stored in the freezer at −20 °C.

### 2.1. Determining Hormone Concentrations

Estradiol (E2) and follicle-stimulating hormone (FSH) concentrations were determined from the blood serum. The total concentration of these hormones was determined by means of the electrochemiluminescence (ECLIA) method with monoclonal antibodies using a COBAS E411 (Roche Diagnostics, Poland) analyzer. The sensitivity limit for FSH was 0.10 mlU/mL, while for E2 it was 5.0 pg/mL.

### 2.2. Data Collection

Genomic DNA from peripheral blood leukocytes was extracted using a High Pure PCR Template Preparation extraction kit (Roche Diagnostics, Mannheim, Germany). The extraction was performed according to the manufacturer’s instructions. The genetic investigation was carried out in a laboratory of the Department of Infectious Tropical Diseases at the Pomeranian Medical University, Szczecin, Poland. DNA samples were stored at 4 °C for further analysis.

### 2.3. Genotyping of the Rs7799039 Mutation of the LEP Gene

Analysis of the *LEP*-2548 G>A (rs7799039) polymorphism was carried out using polymerase chain reaction–restriction fragment length polymorphism (PCR–RFLP). The following primers were used: forward 5′-TTTCCTGTAATTTTCCCGTGA-3′ and reverse 5′-AAAGCAAAGACAGGCATAAA-3′. The PCR time and temperature profile were as follows: initial denaturation at 94 °C for 5 min then 38 cycles of denaturation at 94 °C for 20 s, annealing at 54 °C for 40 s, extension at 72 °C for 40 s, and final extension at 72 °C for 8 min. The 242 base pair (bp) amplicon was digested with 1 unit of *CfoI* restriction enzyme for 24 h at 37 °C and resolved on 3% agarose gel stained with GelStarTM (Lonza, Rockland, ME, USA) for visualization under UV light. Wild allele (-2548 G) was cleaved into 181 and 61 bp restriction fragments, and the mutant one (-2548 A) remained unleaded. Random samples were genotyped twice, independently. All results were found to be reliable.

### 2.4. Genotyping of the Rs429358 and Rs7412 Mutations of the APOE Gene

Genotypes were determined by real-time PCR using the StepOne Real-Time PCR System (Applied Biosystems, Foster City, CA, USA) and TaqMan SNP Genotyping Assays for two *APOE* polymorphisms, including rs429358 and rs7412 (Life Technologies, Assay ID: C_3084793_20, C_904973_10, respectively; Foster City, CA, USA). The data were analyzed using Taq Man Genotyper Software v. 1.0.1 (Applied Biosystems, Foster City, CA, USA).

## 3. Statistical Analysis

Data analysis was performed using SPSS Statistics v. 17.0 software (SPSS Inc., Chicago, IL, USA). Basic statistics included determination of group sizes, minima, maxima, arithmetic means, and standard deviations. The normality of the distributions was tested using a Shapiro–Wilk test. The quantitative data was analyzed using the Mann–Whitney *U*-test, Student’s *t*-test, and the Kruskal–Wallis test, as well as ANOVA. Data measured on a dichotomous scale were analyzed using a chi-square test of independence with Yates’ correction. The results were deemed significant at *p* < 0.05.

### Permission to Conduct Research

The study was approved by the Bioethics Committee of the Pomeranian University, Szczecin, with approval number BN-001/98/98.

## 4. Results

A total of 252 postmenopausal women (Table 1) were enrolled in the study and divided into three groups based on the time since their final menstruation: women up to 5 years post-menopause (Group A; *n* = 88), women 6–10 years post-menopause (Group B; *n* = 81), and women over 10 years post-menopause (Group C; *n* = 83). The analysis of genotype frequencies (Table 2) showed that the most frequent polymorphic variant of the *APOE* gene was *APOE3E3* (70.45% in Group A, 77.78% in Group B, 69.88% in Group C). In the case of polymorphism -2548 G>A of the *LEP* gene, the AG genotype was dominant (52.27% in Group A, 48.15% in Group B, 60.24% in Group C).

By analyzing the groups (Table 3), it was observed that people with the *APOE3/E3* genotype entered menopause insignificantly later than patients with the other genotypes. Additionally, in the group of patients with the *APOE3E3* genotypes, differences in the E2 concentration were significantly related to the period of time since their final menstruation (*p* = 0.04). Among these patients, a higher concentration was observed in Group B than in Groups A and C. In women with different *APOE* genotypes, this relation was also not found (*p* = 0.23). While analyzing the influence of the *APOE* genotype on body mass index (BMI), the FSH concentration, and the age of first menstruation, statistical significances were not observed.

The analysis of the relation between the -2548 G>A *LEP* polymorphism and the time of onset of menopause, BMI values, FSH and E2 concentrations, and first menstruation age did not show any significant differences.

## 5. Discussion

In the context of such a complex issue as the perimenopause in women, researchers have chosen a variety of genetic studies with which to identify the biological determinants of menopause. This is not an easy task, however, because menopause is multigenic and multifactorial. Nevertheless, researchers have reported their attempts to combine genetic variants with different aspects of menopausal syndromes [3,5,6,32]. We selected two genes in our study: one for *APOE* and one for *LEP*, on the basis of biological conditions mentioned in [33,34,35,36,37,38] to be associated with menopause.

In our study of the *APOE* gene (rs429358 and rs7412), in Groups A and C, a lower estradiol concentration was found in women with the E3E3 genotype than in the case of the other genotypes (*E2E2, E2E3, E2E4, E3E4, E4E4*). However, these results were not statistically significant. It should be noted that mean estradiol for each group is close to detection limit, which suggests that a substantial proportion of the study samples fall below the limit of the assay. The power analysis demonstrated that the power test was almost 20% (0.18). With age, the *E3E3* genotype women are more prone to have a lower estradiol concentration than women with other genotypes in the polymorphism investigated here. When the age of the patients who took part in the study was considered, this relation was not observed. This is evidence that this relation is not directly dependent on the patient’s age, but rather depends on the age when menopause occurred.

The *APOE* gene and its polymorphic variants investigated here are described in the literature primarily as genes predisposed to Alzheimer’s disease. In the study of Yuan *et al.* [32], apolipoprotein E and oxidative damage were correlated with the risk of Alzheimer’s. Those researchers found that plasma and erythrocyte antioxidant parameter levels were associated with *APOE* rs429358, rs7412 polymorphism. The influence of the *APOE* rs429358 polymorphism on plasma and erythrocyte antioxidant parameters could be modified by the GSTT1 genotype; the influence of *APOE* rs7412 could be modified by the GSTM1 (genes for the glutathione S-transferase family) genotype.

Both female sex [33] and the *E4* variant of the *APOE* coding gene are among the most important and best-known risk factors in Alzheimer’s [39]. Researchers have identified a protective effect of estrogens in the context of this disease’s occurrence in women [34]. In our study, the estradiol concentration for the *E2E2, E2E3, E2E4, E3E4*, and *E4E4* genotypes compared to *E3E3* genotype was higher in the Group A and Group C age ranges after menopause. However, it should be emphasized that the *E3E3* genotype is very common, so this group has a larger sample size. Perhaps the “other genotypes” group might not show this significant difference because the sample size of this group is smaller. Furthermore, Lambrinoudaki *et al.* [35] observed in their pilot study that polymorphisms of *APOE* and paraoxonase 1 are associated with different levels of the thyroid hormone and anti-TG antibody levels in the study population. A statistically significant correlation was found between apolipoprotein E polymorphisms and serum thyroid hormones: carriers of the E2 or E4 allele of the *APOE* gene had lower levels of FT4 than women with the *E3E3* genotype. A statistically significant positive association was also observed between anti-TG antibodies and the presence of the E2 allele of the *APOE* gene.

Apolipoprotein E [40,41] (*APOE*) is a 34-kDa protein that plays an important role in lipoprotein metabolism by association with lipoprotein particles and with members of the low-density lipoprotein (LDL) receptor family. It is known that there are three *APOE* isoforms (E2, E3, and E4), each of which have different affinities for their binding receptors. *APOE2* has the defective characteristic of binding to low-density lipoprotein receptor (LDLR) through the cysteine at amino acid position 158, which affects the upregulation of the synthesis of 3-hydroxy-3-methylglutaryl coenzyme A (HMG-CoA) and LDLR and ultimately results in low serum LDL levels [42]. Lee D.J. *et al.* [36], in their study of *APOE3*, suggest that obesity or metabolic syndrome risk should be effectively managed in *APOE3* isoform groups to reduce serum LDL in postmenopausal Korean women. In a different study, Issa *et al.* [37] found that menopause is associated with changes in lipid levels, resulting in an increased risk of atherosclerosis and cardiovascular events. They noticed that medication used to lower the level of cholesterol (Atorvastatin) downregulates the *APOE* mRNA expression and is modified by *APOE* genotypes in peripheral blood mononuclear cells from postmenopausal women.

A number of researchers have associated *LEP* and polymorphisms of its gene with changes that take place in physiological processes in women after menopause. Lee *et al.* [38] studied the following polymorphisms of the *LEP* gene in 592 postmenopausal Korean women: c.280 G>A, *LEPR* c.326 A>G, *LEPR* c.668 A>G, *LEPR* c.1968 G>C, *LEPR* c.2096 C>T, *ADRB2* c.46 A>G, *ADRB2* c.79 C>G, *ADRB2* c.718 T>C, ADRB2 c.741 G>T, *ADRB2* c.769 G>A, and *ADRB3* c.190 T>C. Serum levels of leptin, soluble leptin receptor, osteoprotegerin, bone alkaline phosphatase, and carboxy-terminal of type I collagen were measured, and the bone mineral density at the lumbar spine and femoral neck were also examined. Among the polymorphisms measured, only *LEPR* c.1968 G>C was found to be associated with bone mineral density at the femoral neck, and higher BMI values were observed with an increasing number of G alleles. Osteoporosis at the femoral neck was 3.27 and 3.89 times more frequently observed in the AG and GG genotypes than in the AA genotype with the ADRB2 c.46 A>G polymorphism. However, no significant differences in serum levels of leptin, soluble leptin receptor, free leptin index, osteoprotegerin, or bone turnover markers were detected among single or haplotype genotypes.

There is no association found in the literature between the polymorphism of the *LEP* gene studied here and hormones in women after menopause. Our research study is thus pioneering in this respect. No associations with the *LEP* gene or its genotypic or allelic variants were observed in the groups under study.

## 6. Conclusions

To date, attempts to formulate a correct model of the association between E2 and FSH concentration with polymorphisms of various genes of menopause in women have not been successful. This relationship is difficult to study because of the number of nongenetic factors involved. Environmental factors can explain changes in hormone levels in postmenopausal women. Plans are therefore being made for further studies involving a larger group of patients.

## Figures and Tables

**Table 1:** Baseline characteristics of study participants.

Parameters	Group A: Women up to 5 Years Post-Menopause (*n* = 88)				Group B: Women 5–10 Years Post-Menopause (*n* = 81)				Group C: Women 10–15 Years Post-Menopause (*n* = 83)			
	$\bar{X}$	SD	Min	Max	$\bar{X}$	SD	Min	Max	$\bar{X}$	SD	Min	Max
Age (years)	54.8	3.6	47.0	63.0	57.5	4.2	41.0	67.0	65.2	6.0	53.0	78.0
Age of menopause (years)	52.0	3.2	44.0	60.0	49.8	3.9	35.0	58.0	48.6	4.3	33.0	57.0
Last menstrual period (years)	2.8	1.5	1.0	5.0	7.7	1.4	6.0	10.0	16.6	5.9	11.0	45.0
Height (m)	1.6	0.1	1.5	1.7	1.6	0.1	1.5	1.7	1.6	0.1	1.5	1.7
Weight (kg)	71.1	11.4	52.0	103.0	68.6	9.9	51.0	100.0	68.7	11.4	45.0	99.0
BMI (kg/m^2^)	27.4	4.4	21.1	39.45	26.3	3.8	20.5	37.6	26.4	4.0	18.0	36.8
FSH (mIU/mL)	70.2	26.4	26.1	146.1	74.2	24.8	30.0	145.8	76.7	24.9	28.0	146.2
E2 (pg/mL)	9.0	5.7	5.0	21.3	7.6	5.1	5.0	23.4	7.7	4.8	5.0	19.4
Number of children born	1.6	0.8	0	3	1.9	0.9	0	4	1.8	0.8	0	4
Age at first birth (years)	24.2	3.9	18.0	38.0	23.6	3.0	18.0	34.0	24.1	3.4	19.0	36.0
Age of menarche (years)	13.5	1.8	10.0	19.0	13.7	1.9	9.0	18.0	14.3	1.5	10.0	18.0

BMI: body mass index; FSH: follicle-stimulating hormone; E2: estradiol; $\bar{X}$: arithmetic mean; SD: standard deviation.

**Table 2:** Frequency of particular genotypes of the *APOE* and *LEP* genesin study groups.

Genotypes		Group A Women up to 5 Years Post-Menopause (*n* = 88)		Group B Women 5–10 Years Post-Menopause (*n* = 81)		Group C Women 10–15 Years Post-Menopause (*n* = 83)	
**Gene**	Genotype	Frequency	% of *n*	Frequency	% of *n*	Frequency	% of *n*
*APOE*	E2E2	0	0.00	0	0.00	2	2.41
	E2E3	9	10.23	9	11.11	7	8.43
	E2E4	0	0.00	2	2.47	1	1.20
	E3E3	62	70.45	63	77.78	58	69.88
	E3E4	16	18.18	7	8.64	13	15.66
	E4E4	1	1.14	0	0.00	2	2.41
*APOE*	Other genotypes	26	29.55	18	22.22	25	30.12
	E3E3	62	70.45	63	77.78	58	69.88
*LEP*	AA	13	14.77	13	16.05	12	14.46
	GA	46	52.27	39	48.15	50	60.24
	GG	29	32.95	29	35.80	21	25.30
*LEP*	Other genotypes	42	47.73	42	51.85	33	39.76
	GA	46	52.27	39	48.15	50	60.24

**Table 3:** Statistical analysis of parameters in postmenopausal women, including polymorphic variant *APOE*.

*APOE*	Group	Other Genotypes (*E2E2, E2E3, E2E4, E3E4, E4E4*)					E3E3					*p* **
		$\bar{X}$	SD	Min	Max	*p* *	$\bar{X}$	SD	Min	Max	*p* *	
Age of menopause (years)	A	51.3	3.6	46.0	58.0	<0.01	53.2	3.1	46.0	58.0	<0.01	0.70
	B	48.5	2.4	45.0	52.0		50.0	2.8	46.0	58.0		0.46
	C	49.0	3.3	42.0	53.0		49.0	4.9	33.0	57.0		0.76
BMI (kg/m^2^)	A	27.4	4.5	22.3	36.4	0.91	26.5	4.0	21.3	37.5	0.31	0.82
	B	27.2	5.4	22.9	37.6		26.2	2.6	21.5	31.2		0.72
	C	27.2	4.4	22.0	33.9		25.7	3.8	18.0	34.1		0.72
FSH (mIU/mL)	A	58.4	18.5	26.2	94.1	0.16	66.0	24.5	28.0	124.6	0.75	0.22
	B	64.3	19.6	35.8	91.4		68.6	25.3	30.0	129.7		0.79
	C	75.2	32.5	37.2	140.9		78.3	19.7	48.8	136.4		0.55
E2 (pg/mL)	A	10.2	6.0	5.0	20.1	0.23	8.6	5.2	5.0	21.3	**0.04**	0.35
	B	7.1	3.0	5.0	23.4		7.5	5.7	5.0	23.3		0.95
	C	8.4	6.4	5.0	19.4		5.9	3.9	5.0	15.1		0.09
Age of menarche (years)	A	13.4	1.8	10.0	16.0	0.23	13.6	1.9	11.0	19.0	0.15	0.88
	B	12.7	3.1	9.0	18.0		14.0	1.4	11.0	16.0		0.11
	C	14.3	1.6	12.0	18.0		14.3	1.5	10.0	17.0		0.57

Group A included women up to 5 years post-menopause, Group B included women 5–10 years post-menopause, and Group C included women over 10 years post-menopause.; *p* *: statistical significance between Groups 1, 2, and 3 within the genotypes under study; *p* **: statistical significance between the E3E3 genotype and the remaining genotypes in the group of patients under study (*E2E2, E2E3, E2E4, E3E4, E4E4*); BMI: body mass index; FSH: follicle-stimulating hormone; E2: estradiol; $\bar{X}$: arithmetic mean; SD: standard deviation.
